# Behavioral and Socioeconomic Risk Factors Associated with Probable Resistance to Ceftriaxone and Resistance to Penicillin and Tetracycline in *Neisseria gonorrhoeae* in Shanghai

**DOI:** 10.1371/journal.pone.0089458

**Published:** 2014-02-19

**Authors:** Molly A. Trecker, Cheryl Waldner, Ann Jolly, Mingmin Liao, Weiming Gu, Jo-Anne R. Dillon

**Affiliations:** 1 Vaccine and Infectious Disease Organization – International Vaccine Centre, Saskatoon, Saskatchewan, Canada; 2 School of Public Health, University of Saskatchewan, Saskatoon, Saskatchewan, Canada; 3 Western College of Veterinary Medicine, University of Saskatchewan, Saskatoon, Saskatchewan, Canada; 4 Centre for Infectious Disease Prevention and Control, Health Canada and Public Health Agency of Canada, Ottawa, Ontario, Canada; 5 Department of Microbiology and Immunology, College of Medicine, University of Saskatchewan, Saskatoon, Saskatchewan, Canada; 6 Shanghai Skin Disease and Sexually Transmitted Disease Hospital, Shanghai, China; Institut National de la Recherche Agronomique, France

## Abstract

Globally, incidence of *Neisseria gonorrhoeae* infection is once again the highest of the bacterial sexually transmitted infections. The bacterium can produce serious complications in those infected, and emerging resistance to third generation cephalosporins could usher in an era of potentially untreatable gonorrhea. This research aimed to identify risk factors for antibiotic resistant gonorrhea infection among clients at a Shanghai sexually transmitted infection clinic over two time periods, 2004–2005 and 2008–2011. Demographic and risk factor behavior data, and biological samples for antimicrobial resistance analysis, were collected. Statistical models were built to identify risk factors associated with probable resistance to ceftriaxone and resistance to penicillin and tetracycline. High levels of ciprofloxacin resistance (98%) in our sample precluded examining its risk factors; all isolates were susceptible to spectinomycin. Overall (P<0.001), chromosomal (P<0.001), and plasmid-mediated (P = 0.01) penicillin resistance decreased from the first to second period of the study. For tetracycline, chromosomal resistance decreased (P = 0.01) and plasmid-mediated resistance increased (P<0.001) between the first and second periods of study. In multi-level multivariable regression models, male gender (P = 0.03) and older age (P = 0.01) were associated with increased minimum inhibitory concentrations to ceftriaxone. Male gender (P = 0.03) and alcohol use (P = 0.02) were associated with increased odds of overall tetracycline resistance. Male gender was associated with increased odds of chromosomally-mediated tetracycline resistance (P = 0.04), and alcohol use was associated with increased odds of plasmid-mediated tetracycline resistance (P = 0.02). Additionally, individuals in middle-salary categories were found to have lower odds of plasmid-mediated resistance to tetracycline compared with those in the lowest salary category (P≤0.02). This study is one of the first to use multilevel analysis to consider the association between risk factors for gonorrhea infections and mechanisms of resistance to individual antibiotics. Such information is urgently needed to combat the growing threat of untreatable gonorrhea.

## Introduction

Infections caused by *Neisseria gonorrhoeae* have afflicted human beings for centuries. The organism causes localized infections of the throat, rectum, or urogenital tract and has the potential for serious complications including pelvic inflammatory disease and ectopic pregnancy in women as well as infertility in both sexes [1]. Transmission to newborns can lead to serious complications including blindness. Infection with gonorrhea also facilitates HIV transmission [2]. Gonorrhea has become, once again, the most commonly transmitted bacterial sexually transmitted infection (STI) globally [3]; it is estimated that 106.1 million cases occur annually around the world [3]. Because gonorrhea infection is often asymptomatic, the true burden of disease is likely much higher.

While gonorrhea has generally been effectively treatable with a single antibiotic dose, drug resistance has emerged to each class of therapeutic agent introduced since the 1940s. Recently, treatment failures to the last-line recommended drugs – third generation cephalosporins – have been reported globally [4]–[7]. The threat of untreatable gonorrhea [8] poses a significant public health challenge both through its associated effects on fertility, birth outcomes, and HIV transmission rates, as well as the potential for increased transmission and complications of infections. Identification of factors associated with antimicrobial resistance (AMR) could help combat this growing epidemic by informing policies around antimicrobial use and targeting intervention programs to those at heightened risk for AMR infection. Behavioral and socioeconomic factors are well known to affect STI transmission [9]–[12]. More research is needed to understand the influence of socioeconomic and behavioral factors on the resistance of *N. gonorrhoeae* isolates to specific antibiotics such as ceftriaxone, including their mechanisms of resistance.

Research into antibiotic resistant gonorrhea infections is particularly relevant in China, where years of over-the-counter antibiotic availability, coupled with poor antibiotic management and prescribing practices, have resulted in widespread antimicrobial resistance [13]. We examined the influence of demographic factors as well as previous STIs, use of over the counter antibiotics, and risky sexual practices on the probability of gonorrhea infection with reduced susceptibility or probable resistance to ceftriaxone, or resistance to penicillin or tetracycline. These analyses are valuable in identifying characteristics that could be used to better target interventions based on risk factor profiles, potentially reducing the need for complex and expensive susceptibility testing in resource limited regions. We combine epidemiologic and previously published biologic data in multi-level analyses for the first time to identify behaviors and client attributes that are associated with an increased risk of infection with AMR *N. gonorrhoeae* isolates.

## Methods

### Sample

Epidemiologic and demographic information were obtained from two cross-sectional samples of symptomatic male patients who tested positive for gonorrhea at the Shanghai Sexually Transmitted Infection and Skin Disease Hospital during 2004–2005 and 2008–2011. Partners brought in for evaluation and who consented to participate in the study were also included. Study design and survey descriptions for the first phase of the study (n = 483 primary interviews) have been previously published; the second phase (n = 299 primary interviews) followed a similar structure [14]–[18]. The analysis presented here included a convenience subsample of 384 cases with complete antimicrobial resistance data.

### Epidemiologic Data

Staff at the Shanghai Skin Disease and STD Hospital collected information on demographic variables including gender, birthdate, salary, residency status in Shanghai, district of residence, and level of education attained, as well as STI history, history of antibiotic use, sexual practices, number of partners, and use of alcohol and drugs. Membership in a cluster was abstracted to account for pairs or triples present in the dataset, which arose when any index patient subsequently brought one or more partners to treatment.

### Isolate Identification and Antimicrobial Susceptibility Determination

The collection, isolation, and identification of *N. gonorrhoeae* isolates has been described previously, as have methods for the determination of minimum inhibitory concentrations (MICs) to ceftriaxone, penicillin, tetracycline, ciprofloxacin, and spectinomycin [18], [19]. β-lactamase production was determined using nitrocefin and MIC breakpoints were those recommended by the Clinical and Laboratory Standards Institute (CLSI) [19]. MIC data were retrieved from data reported earlier [15]–[17].

### Statistical Analysis

Two MIC breakpoints were used to explore risk factors for reduced susceptibility (0.03 µg/mL) [20]–[22] or probable resistance (0.125 µg/mL) [23] to ceftriaxone; separate models were constructed for each breakpoint. The World Health Organization classifies isolates of *N. gonorrhoeae* with ceftriaxone MICs of ≥0.125 as representing “probable resistance” [23]. Two different outcomes were examined for penicillin and tetracycline: overall resistance versus susceptibility, and mechanism of resistance – plasmid-mediated and chromosomally-mediated – versus susceptibility. For penicillin, any isolate with an MIC value <2.0 µg/mL was classified as susceptible [19] and all other isolates were classified as resistant. Of these resistant isolates, any that were β-lactamase positive were classified as having plasmid-mediated resistance while all others were classified as having chromosomally-mediated resistance [19]. For tetracycline, any isolate with an MIC value <2.0 µg/mL was classified as susceptible, while all others were classified as resistant [19]. A breakpoint of 16 µg/mL was used to separate chromosomally-mediated resistance (≥2 and <16 µg/mL) from plasmid-mediated resistance (≥16 µg/mL) [19].

Data were managed in Microsoft Excel and Microsoft Access, while all statistical analyses were performed using Stata IC/12.1 [24]. Comparisons of demographic characteristics of participants in Phase 1 and Phase 2 were made using Chi-square and Mann-Whitney U tests. To identify predictors of AMR infection, multi-level regression models were built using the gllamm program for generalized linear mixed models (GLMM) in Stata IC/12.1 [24]. Six individual models were built to investigate the following outcomes: reduced susceptibility to ceftriaxone at 0.03 µg/ml, probable resistance to ceftriaxone at 0.125 µg/ml, resistance to penicillin, mechanism of penicillin resistance, resistance to tetracycline, and mechanism of tetracycline resistance.

We used logistic regression to investigate the presence or absence of reduced susceptibility or resistance based on MIC data, and multinomial regression models to examine mechanisms of resistance [25]. The analysis was limited to relevant independent variables with frequencies >5% and <95%, which resulted in a set of 11 variables to be tested (Table 1). Phase of study was also initially included as a variable in each model. The continuous predictor variables age and salary were categorized into quintiles for analysis: 14–26 years (n = 86), 27–31 years (n = 73), 32–37 years (n = 69), 38–45 years (n = 76), and 46–83 years (n = 74) and (in Chinese Yuan) 0–1200 (n = 85), 1300–2000 (n = 70), 2200–3500 (n = 84), 4000–5500 (n = 69), and 6000–200000 (n = 74).

**Table 1:** Selected demographic characteristics and risk behaviors of a sample of clients from the Shanghai Sexually Transmitted Infection and Skin Disease Hospital (n = 384).

	Mean (SD), Median (IQR), or N (%)		
Variable	Phase 1 (n = 189)	Phase 2 (n = 195)	Overall
Mean age (years)^1^	34.5 (10.0)	36.7 (12.1)	35.6 (11.2)
Median salary (Yuan)*	2500 (1200,4500)	3500 (2000,5500)	3000 (1500,5000)
Gender†			
* Male*	156 (82.5)	180 (92.3)	336 (87.5)
* Female*	33 (17.5)	15 (7.7)	48 (12.5)
Education level			
* None/less than primary*	5 (2.6)	2 (1.0)	7 (1.8)
* Primary/middle*	61 (32.2)	59 (30.3)	120 (31.3)
* High school*	63 (33.3)	49 (25.1)	112 (29.2)
* Above high school*	60 (31.7)	85 (43.6)	145 (37.8)
Previous STI history	69 (36.5)	70 (35.9)	139 (36.2)
Previous bacterial STI*	31 (16.4)	62 (31.8)	93 (24.2)
Wash genitals before or after sex* ^2^	121 (64.0)	165 (84.6)	286(74.5)
Take OTC ABX ever* ^3^	17 (9.0)	37 (19.0)	54(14.1)
Use alcohol during sex ever^4^	76 (40.2)	67 (34.4)	143(37.3)
Use drugs during sex ever^5^	10 (5.3)	18 (9.2)	28(7.3)
Number of partners in previous 3 months^6^			
* One*	82 (43.4)	68 (34.9)	150 (39.1)
* Two*	50 (26.5)	66 (33.8)	116 (30.2)
* Three or more*	45 (23.8)	49 (25.1)	94 (24.5)

*Significant difference between phases at p<0.001.

† Significant difference between phases at p = 0.004.

1 6 missing ^2^11 missing ^3^74 missing ^4^1 missing ^5^2 missing ^6^24 missing.

Intra-class correlation coefficients (ICC) were calculated to determine the amount of variability in the null models for each of the six outcomes accounted for by testing phase of study, membership in a dyad in the dataset, and district of residence as random effects [26]. The null models with the lowest Akaike Information Criterion (AIC) values were selected for building the final models, all of which included district as a random intercept. If <3% of observations were missing for a particular covariate, listwise deletion was used; otherwise, non-responses were considered as a unique response category.

Only potential risk factors unconditionally associated with the outcome (p≤0.3) were considered as candidates for inclusion in multivariable models (Table S1). Manual backwards elimination was used to build the final model for each outcome. Only significant independent risk factors (p<0.05) and potential confounders for each outcome, including age, gender, and study phase, were retained in the final multivariable models. Confounding was recognized when the difference between crude odds ratio for a risk factor-outcome association of interest and the same odds ratio adjusted for the potential confounder was >10%. After establishing main effects models for each outcome, all possible two-way interactions were considered; only interactions significant at p<0.05 were retained in the final models.

Intraclass Correlation Coefficients (ICCs) were estimated as σ^2^_district_/(σ^2^_district_+π^2^/3) for each final model [27]. Plots of standardized residuals were examined for each model to check for outliers.

### Ethics Statement

Ethical approval for this study was obtained from the Ottawa Hospital Research Ethics Board, the Ethics Committee of the Shanghai Municipal Bureau of Public Health, and the University of Saskatchewan Biomedical Research Ethics Board. Written consent was obtained from all participants. Minors under the age of 18 provided their own consent to participate as they are regarded as emancipated when they take responsibility for seeking reproductive health care. All participants were treated according to Canadian and Chinese STI standard recommendations; no extra procedures were given, other than the time to complete the questionnaire.

## Results

### Summary of the Study Population and AMR Findings

Antimicrobial susceptibility data were available for 189 *N. gonorrhoeae* isolates from the first phase of the study and 195 from the second phase for a total of 384 isolates; 337 isolates were from index cases and 47 were from partners brought to treatment. The participants came from 19 different districts of Shanghai; the mean number of cases per district was 11.3 with a range of 1 to 57. Only 16 (4.2%) reported being residents of Shanghai for fewer than 6 months.

There was no significant difference in mean age, education level, previous STI history, use of alcohol or drugs during sex, or reported number of partners in the previous three months between participants from the two study phases. Clients ranged in age from 14 to 83, with a mean age of 35.6 years; 6 clients did not disclose their age. The majority (67.0%) of participants had at least a high school education, and 36 percent of cases reported having had at least one previous STI. Just over one-third (37.3%) of participants reported using alcohol during sex (1 missing), and 7.3% reported using drugs during sex (2 missing). Slightly over half (54.7%) of participants indicated they had 2 or more sexual partners in the last 3 months, and 39.1% said they had only one partner in that time (24 individuals did not respond). (Table 1).

For five variables, there were significant differences between phases. Median salary over the previous 3 months reported by Phase 2 participants was higher (3500 Yuan) than that reported by Phase 1 participants (2500 Yuan) (p≤0.001). In the sample population overall, most cases were male (87.5%); 12.5% of cases were female partners subsequently brought to treatment. However, female partners comprised 17.5% of the Phase 1 population and only 7.7% of the Phase 2 population (p = 0.004). A higher proportion (31.8%) of participants in Phase 2 as compared to Phase 1 (16.4%) reported having had a previous bacterial STI (p≤0.001), and 84.6% of Phase 2 participants compared to 64% of Phase 1 participants indicated that they washed their genitals before and/or after intercourse (p≤0.001); 11 did not respond. Lastly, 19% of Phase 2 participants and 9% of Phase 1 participants indicated previous use of over the counter antibiotic agents (p≤0.001) (data missing for 74 individuals or 19.3%). (Table 1).

Ceftriaxone MICs in our sample ranged from 0.004 to 0.25 µg/mL (Table 2). The majority of isolates (75.8%) had reduced susceptibility to ceftriaxone at the 0.03 µg/mL breakpoint, and 10.9% showed probable resistance at the 0.125 µg/mL breakpoint (Table 3). Penicillin MICs ranged from 0.125 to 64 µg/mL (Table 2). Based on CLSI MIC breakpoints [19], overall 13.5% of isolates were classified as susceptible to penicillin and 86.5% were resistant over the two time periods tested (Table 3). Chromosomally-mediated resistance to penicillin was identified in 36.2% of cases overall, and 50.3% had a gonorrhea isolate with plasmid-mediated penicillin resistance (Table 3). Tetracycline MICs ranged from 0.06 to 64 µg/m (Table 2). Tetracycline resistance was present in 54.7% of isolates overall and 45.3% were susceptible based on MIC data (Table 3). Chromosomally-mediated resistance to tetracycline was exhibited by 23.7% of isolates overall and plasmid-mediated resistance was found in 31% (Table 3).

**Table 2:** MICs of 384 *N. gonorrhoeae* isolates from Shanghai to ceftriaxone, ciprofloxacin, spectinomycin, penicillin, and tetracycline.

	MIC N (cumulative %)														
Antibiotic	*0.004*	*0.008*	*0.016*	*0.03*	*0.06*	*0.125*	*0.25*	*0.5*	*1*	*2*	*4*	*8*	*16*	*32*	*64*
*Ceftriaxone*	3 (0.8)	24 (7.0)	66 (24.2)	133 (58.9)	116 (89.1)	40 (99.5)	2 (100)	–	–	–	–	–	–	–	–
*Ciprofloxacin* 1	–	–	1 (0.3)	–	1 (0.5)	–	–	4 (1.6)	10 (4.2)	40 (14.6)	69 (32.6)	121 (64.2)	103 (91.1)	32 (99.5)	2 (100)
*Spectinomycin*	–	–	–	–	–	–	–	–	–	2 (0.5)	17 (4.9)	44 (16.7)	159 (47.8)	150 (96.9)	12 (100)
*Penicillin*	–	–	–	–	–	1 (0.3)	7 (2.1)	12 (5.2)	32 (13.5)	67 (31.0)	48 (43.5)	22 (49.2)	9 (51.6)	18 (56.3)	168 (100)
*Tetracycline*	–	–	–	–	4 (1.0)	20 (6.3)	31 (14.3)	35 (23.4)	84 (45.3)	59 (60.7)	23 (66.7)	9 (69.0)	26 (75.8)	40 (86.2)	53 (100)

1 N = 383 for ciprofloxacin.

Distribution of MICs for 384 *N. gonorrhoeae* isolates to ceftriaxone, ciprofloxacin, spectinomycin, penicillin, and tetracycline. Number of isolates and cumulative percent for each MIC breakpoint are shown for each antibiotic.

**Table 3:** Prevalence of resistance or reduced susceptibility to ceftriaxone, penicillin, and tetracycline in a sample of clients from the Shanghai Sexually Transmitted Infection and Skin Disease Hospital.

	Phase 1 N (%)	Phase 2 N (%)	Overall$ N (%)
**Total isolates available**	189	195	384
**Ceftriaxone**			
*Reduced susceptibility at MIC≥0.03 µg/ml*	143 (75.7)	148 (75.9)	291 (75.8)
*Probable resistance at MIC≥0.125 µg/ml* †	21 (11.1)	21 (10.8)	42 (10.9)
**Penicillin**			
*Susceptible(MIC<2 µg/ml)* *	14 (7.4)	38 (19.5)	52 (13.5)
*Resistance overall*	175 (92.5)	157 (80.5)	332 (86.5)
*Chromosomal resistance*	78 (41.3)	61 (31.3)	139 (36.2)
*Plasmid-mediated resistance*	97 (51.3)	96 (49.2)	193 (50.3)
**Tetracycline**			
*Susceptible(MIC<2 µg/ml)* *	90 (47.6)	84 (43.1)	174 (45.3)
*Resistance overall*	99 (52.4)	111 (56.9)	210 (54.7)
*Chromosomal resistance*	62 (32.8)	29 (14.9)	91 (23.7)
*Plasmid-mediated resistance*	37 (19.6)	82 (42.1)	119 (31.0)

† WHO 2012.

*CLSI 2009;

$ Phases 1 and 2 combined.

The range of MIC values for each antibiotic were as follows: ceftriaxone 0.004–0.25, penicillin 0.125–64, tetracycline 0.06–64.

Because all isolates were susceptible to spectinomycin (MIC range: 2–64 µg/mL) and 98.2% were resistant to ciprofloxacin (MIC range: 0.016–64 µg/mL) (Table 2), associations of socio-demographic variables and resistance were not explored for these antibiotics.

### Unconditional or Crude Analysis of Predictors for AMR

Level of education, salary, male gender, having reported using drugs during sex, and number of partners reported were unconditionally associated with reduced susceptibility to ceftriaxone at the 0.03 µg/mL breakpoint (Table S1); taking over the counter antibiotics, age, male gender, and drug use were unconditionally associated with reduced susceptibility at the 0.125 µg/mL breakpoint. Phase of study, level of education, having reported a previous bacterial STI, practicing genital washing before or after sex, and taking over the counter antibiotics were associated with overall penicillin resistance; the same variables, along with age and salary, were unconditionally associated with the mechanism of penicillin resistance (Table S1). Level of education, salary, previous STI, previous bacterial STI, male gender, and reported alcohol use during sex were unconditionally associated with overall tetracycline resistance (Table S1). Phase of study, level of education, salary, previous bacterial STI, male gender, and alcohol use were unconditionally associated with the mechanism of tetracycline resistance exhibited (Table S1).

### Final Models of Factors Associated with Reduced Susceptibility and Probable Resistance to Ceftriaxone

In the final multivariable models for reduced susceptibility to ceftriaxone (Table 4a), men were more likely to be infected with a strain with reduced susceptibility to ceftriaxone with MIC≥0.03 µg/mL (OR = 2.2) than women.

**Table 4:** Results of multivariable analysis for reduced susceptibility and probable resistance to ceftriaxone (odds ratios (OR) and 95% confidence intervals (95% CI)).

a.	Ceftriaxone reduced susceptibility (MIC≥0.03 µg/mL) (n = 378)					
Variable	OR	p-value		95% CI		
**Male : Female**	**2.18**	**0.03**		**1.10–4.32**		
Age (4 df)	0.58					
14–26 years	Reference category					
27–31 years	0.72	0.40		0.34–1.53		
32–37 years	0.63	0.23		0.29–1.34		
38–45 years	0.98	0.95		0.45–2.14		
46–83 years	0.62	0.21		0.29–1.32		
*ICC Residential district:*	*3.6×10* ^−*15*^					
**b.**	**Ceftriaxone probable resistance (MIC≥0.125 µg/mL) (n = 378)**					
**Variable**	**OR**		**p-value**		**95% CI**	
Male : Female	1.64		0.45		0.46–5.84	
**Age (4 df)**	**0.02**					
14–26 years	Reference category					
27–31 years	1.18		0.80		0.35–3.83	
32–37 years	0.79		0.73		0.21–2.99	
38–45 years	1.51		0.47		0.50–4.57	
** 46–83 years**	**3.83**		**0.01**		**1.33–11.01**	
**Take OTC antibiotics (2 df)**	**0.03**					
Do not take OTC antibiotics	Reference category					
Take OTC antibiotics	1.64		0.24		0.71–3.82	
**Take OTC antibiotics (refused to answer)**	**0.25**		**0.03**		**0.07–0.88**	
*ICC Residential district:*	*2.0×10* ^−*23*^					

Final models for reduced susceptibility to ceftriaxone with significant predictors shown in bold.

Participants who were over 46 years of age were almost 4 times more likely than those from age 14–26 to have probable resistance to ceftriaxone (MIC≥0.125 µg/mL) (Table 4b). In addition, those who did not answer the question regarding over the counter antibiotic use were less likely (OR = 0.25) to have probable resistance compared to those who did not report over the counter antibiotic use (Table 4b). Based on the extremely small ICC values, the effect of district on reduced susceptibility or probable resistance to ceftriaxone was not important after accounting for other risk factors.

### Final Models of Factors Associated with Penicillin Resistance

In the final multivariable models for penicillin resistance (Table 5), phase of the study was the only significant predictor. Clients who were in Phase 2 of the study were less likely than those in Phase 1 to be infected with any penicillin resistant strain (OR = 0.31), a strain with chromosomal resistance to penicillin (OR = 0.26), or a strain with plasmid-mediated resistance to penicillin (OR = 0.36). District of residence accounted for 7–8% of the unexplained variance in the penicillin outcomes in the final model (Table 5).

**Table 5:** Results of multivariable analysis of factors associated with resistance to penicillin based on MIC classification and type of penicillin resistance based on molecular data (odds ratios (OR) and 95% confidence intervals (95% CI)).

	Penicillin Resistance (n = 378)			Penicillin Resistance Type (n = 378)					
				*Chromosomal : Susceptible*			*Plasmid : Susceptible*		
Variable	OR	p-value	95% CI	OR	p-value	95% CI	OR	p-value	95% CI
**Phase 2 of the study : Phase 1**	**0.31**	**<0.001**	**0.16–0.62**	**0.26**	**<0.001**	**0.12–0.54**	**0.36**	**0.01**	**0.18–0.73**
	*ICC Residential district: 0.07*			*ICC Residential district for chromosomally-mediated: 0.07*					
				*ICC Residential district for plasmid-mediated*: 0.08					

Phase of study was the only significant predictor of penicillin outcomes.

### Final Models of Factors Associated with Tetracycline Resistance

While phase was not a significant predictor of tetracycline resistance versus susceptibility (Table 6a), the occurrence of plasmid- and chromosomally-mediated resistance to tetracycline did vary based on the phase of the study in the final multivariable model (Table 6b). During Phase 2 of the study, individuals were less likely to carry chromosomally-mediated resistant strains than in Phase 1 (OR = 0.48), but were more likely to carry plasmid-mediated resistant strains (OR = 2.9). The odds of carrying plasmid-mediated resistant strains compared to chromosomally-mediated resistant strains were 5.5 times greater [95% CI 2.96–10.28, p<0.01 (data not shown)] in Phase 2 than in Phase 1.

**Table 6:** Results of multivariable analysis for overall and type of tetracycline resistance (resistance (odds ratios (OR) and 95% confidence intervals (95% CI)).

a.		Tetracycline Resistance (n = 376)									
Variable		OR	p-value			95% CI					
**Male: Female**		**2.12**	**0.03**			**1.06–4.25**					
**Education** (4 df)		**<0.001**									
Less than primary or no education		Reference category									
Primary/middle school		2.23	0.35			0.44–10.39					
High school		1.02	0.98			0.21–5.01					
Above high school		0.76	0.74			0.15–3.78					
**Alcohol use**	**1.69**		**0.02**				**1.08–2.64**				
*ICC Residential district:* 4.4*×*10^−10^											
**b.**		**Tetracycline Resistance Type (n = 376)**									
		***Chromosomal: Susceptible***						***Plasmid : Susceptible***			
**Variable**		**OR**		**p-value**	**95% CI**			**OR**		**p-value**	**95% CI**
**Phase 2 of the study : Phase 1**		**0.48**		**0.01**	**0.27–0.84**			**2.85**		**<0.001**	**1.65–4.91**
**Male: Female**		**2.73**		**0.04**	**1.06–7.05**			1.18		0.70	0.52–2.66
**Alcohol use**		1.68		0.07	0.96–2.93			**1.93**		**0.02**	**1.13–3.32**
**Salary (4 df)**		**0.01**									
<1300 Yuan		Reference category									
1300–2000 Yuan		0.78		0.57	0.33–1.84				0.99	0.98	0.46–2.16
** 2200**–**3500 Yuan**		0.78		0.54	0.35–1.72				**0.28**	**<0.001**	**0.12**–**0.64**
** 4000**–**5500 Yuan**		0.77		0.55	0.33–1.81				**0.34**	**0.02**	**0.14**–**0.81**
6000–200000 Yuan		0.57		0.24	0.22–1.47				0.96	0.91	0.42–2.16
*ICC Residential district for chromosomally-mediated:* 7.6×10^−22^											
*ICC Residential district for plasmid-mediated:* 8.9×10^−22^											

Final models for tetracycline outcomes with significant predictors shown in bold.

Male gender was a significant predictor of overall tetracycline resistance and chromosomal resistance to tetracycline, but not plasmid-mediated tetracycline resistance. The odds of finding overall tetracycline resistance were 2.1 times higher in men than in women (Table 6a); for chromosomal resistance compared to no resistance, the odds were 2.7 times higher in men than women (Table 6b).

Both overall tetracycline resistance (OR = 1.7) and plasmid-mediated resistance to tetracycline (OR = 1.9) were more common among those who reported alcohol use during sex (Table 6a,b).

Study participants who earned between 2200 and 5500 Yuan–the middle salary categories–were less likely than those in the lowest salary category (<1300 Yuan) (OR = 0.3) to carry strains that exhibit plasmid-mediated resistance to tetracycline. The effect of district on tetracycline resistance was not important based on the extremely small ICC values (Table 6 a,b).

## Discussion

This study complements and extends previous work aimed at understanding predictors for AMR in clients with gonorrhea infections [28]–[35]. The focus of this research, however, was to identify risk factors for resistance or reduced susceptibility to three specific antibiotics, and to identify associations between behavioral factors and mechanisms of resistance to penicillin and tetracycline. Further, we employed a rigorous statistical approach in our analysis, allowing us to better understand the association of individual variables to AMR infection in the presence of a complex set of potential risk factors.

Thirty-six percent of participants in this study reported a previous STI, and 55% reported 2 or more sexual partners in the last three months. While these fractions are higher than several previous studies that have considered prior STI infection status and number of sexual partners among Chinese participants [12], [36], [37], studies in other countries have noted similar percentages [38]. Neither of these factors was associated with an increased risk of infection with reduced susceptibility to ceftriaxone or resistance to penicillin or tetracycline in this study. While previous STIs would plausibly predispose individuals to treatment with antibiotics and potential development of resistance, once an individual has cleared the resistant infection and stopped taking the medication his or her risk should return to baseline. The absence of the anticipated association between multiple sexual partners and AMR in the present study could be related to misclassification due to social acceptability bias and the relatively high non-response rate for this question.

Probable resistance to ceftriaxone was most common among those in the older age groups. Cole et al. [30] found a similar effect for cefixime in Europe and Ota et al. [35] found a similar effect for quinolone resistant gonococcal infection in Ontario, Canada. It is possible that this age group is associating with sex partners of a different demographic than those of the younger age groups, or has other risk factors that were not captured in our study. The possibility of increased risk of resistance at older ages could have important implications for prevention policy in Shanghai, especially in this era of rapidly emerging cephalosporin resistance. Additionally, this finding highlights the importance of including a broad range of ages of participants in studies addressing risk factors for AMR.

The findings related to over the counter antibiotic use and probable resistance to ceftriaxone are intriguing. While one might expect to find a relationship between use of antibiotics and AMR infections, we were unable to elucidate such a relationship. The only significant predictor was having failed to provide a response to the question, which cannot be clearly interpreted; this finding is likely related to the relatively high nonresponse rate for this question.

Our results show differences in the prevalence of resistance and type of resistance to penicillin, and to type of tetracycline resistance between the two phases of the study. Because the location of the study clinic moved (within Shanghai) between the two phases of data collection, it is possible that this change over time is the result of unmeasured differences in the populations sampled during the two phases. We were able to control for the influence of the variables listed in Table 1 through our multivariable analysis, indicating that the remaining significance of study phase is likely related to unmeasured factors. Clarification of the causes and monitoring of such apparent temporal changes in resistance mechanisms using serial cross sectional and cohort studies could better inform policies around treatment protocols in Shanghai and help to separate the effects of temporal changes from those related to potential unmeasured demographic shifts.

In contrast to our findings for penicillin and tetracycline, there was no apparent difference in the susceptibility to ceftriaxone between the two time periods. Nevertheless, the levels of reduced susceptibility (75.8% at 0.03 µg/mL) were high, and indicate that continued surveillance is critical. Additionally, the 10.9% overall prevalence of isolates with ceftriaxone MICs≥0.125 µg/mL, which is classified as probable resistance by the World Health Organization [23], calls for an alert to be raised at the regional level.

While there has been much research into the association of men who have sex with men (MSM) and antibiotic resistance [28], [29], [34], [39], we found an independent association of male gender (compared to female) with resistance to tetracycline and reduced susceptibility to ceftriaxone among a sample of men who reported female partners. This could indicate that heterosexual males in Shanghai are at higher risk for tetracycline AMR in general, as well as chromosomally-mediated tetracycline resistance specifically, and ceftriaxone reduced susceptibility compared to females. This finding is consistent with that of Ota et al. [35], who found an unconditional association of quinolone resistant gonorrhea in males as compared to females among a population from Ontario, Canada. Further research exploring male gender as a risk factor for AMR is needed to understand what might influence the apparent association in this population. This is especially important in China because stigma and discrimination against MSM might prevent men from truthfully answering questions about sexual partnerships or serve as barriers to seeking health services [40], potentially complicating the association between male gender and AMR. Additionally, the proportion of MSM who also have sex with females has been shown to be high [41], highlighting the need to better understand this population and potential risk for AMR gonorrhea infections in the population as a whole.

The association between alcohol use and tetracycline resistance, both overall and plasmid-mediated, could suggest that those with riskier behaviors were more prone in this population to acquiring plasmid-mediated tetracycline-resistant gonorrhea infection. Previous studies have shown that alcohol use is positively correlated with risky sexual behaviors such as having multiple sex partners, risky partners, never using condoms, and infection with STIs [42]–[45]. While the relationship between alcohol use and risky sexual practices and STIs is relatively well-established, the association of alcohol use with plasmid-mediated tetracycline resistance is new. This finding must be considered in the absence of observed associations with other more proximate risk behaviors such as use of over the counter antibiotics and high numbers of partners. It is possible that alcohol use served as a marker for other risk behaviors in our study. For example, those using alcohol could be members of a network of individuals who have a higher risk of STIs, are using antibiotics for treatment, and, consequently, are more likely to carry resistant strains. The relationship we found between alcohol and plasmid-mediated tetracycline resistance, specifically, could reflect that the predominant strains being passed through this network at the time of the study had plasmid-mediated tetracycline resistance, given there is no biologically plausible explanation for an association between alcohol use and mechanisms of resistance. At the least, our findings suggest the need to further explore the relationship of such risk behaviors and exposure to AMR in general as well as to *specific* types of AMR strains to clarify this relationship. Such information would be especially useful in settings where individuals do not provide complete or truthful answers to questions about more immediate risk factors.

The finding that plasmid-mediated tetracycline resistance was less common in middle salary categories compared to those in the lowest salary category could be related to socio-demographic factors. However, this relationship was not found for the other outcomes under investigation. There is a possibility that individuals with mid-level incomes are associating with partners with different exposure histories than those with lower incomes. Associations of income category to infections with STIs/HIV have been found in other studies [11], [12]; however, further, targeted research would be needed to explore the association identified in our study.

This study had several limitations. Foremost, the relatively small sample size of participants with characterized *N. gonorrhoeae* isolates, drawn from a population where antimicrobial resistance is extremely prevalent [14], limited the potential to explore outcomes within this dataset to the three antibiotics that exhibited some variability in resistance patterns. The participants were all drawn from the same medical clinic, and had very similar questionnaire responses. Missing data also limited the initial choice of variables to be considered for model building from the more substantial list of the original questions. It is also likely that there was under-reporting of some risk behaviors [46]. Further, because the location of the clinic moved between the two phases of data collection, it is difficult to differentiate temporal shifts in AMR prevalence from other unmeasured differences between the two populations. However, we tested all of the variables listed in Table 1 as part of the model building process, and included in the final model any variables shown to have a confounding effect. This analytical approach controls for the influence of measured differences in the two populations related to these factors on the other variables in the final models. Also, it is likely that the relatively high rate of nonresponse related to antibiotic use contributed to the failure to see an association between over the counter antibiotic use and reduced susceptibility or resistance.

In conclusion, although there was a high prevalence of resistance in the isolates tested [18], we were able to identify several risk factors for infection with gonorrhea with reduced susceptibility or probable resistance to ceftriaxone, resistant to penicillin and tetracycline, and also for the mechanism of resistance to penicillin and tetracycline. The use of multi-level regression analysis to account for potential clustering in the data and explore multinomial outcomes strengthens the study and represents a thorough and sophisticated approach to analysis of such data. We have laid the groundwork for further studies to better understand behavioral factors associated with gonorrhea infection with resistant organisms–knowledge that is central to tailoring effective policies for combatting the emergence of untreatable gonorrhea focused on targeting behavioral risk factors.

## Supporting Information

Table S1 P-values for the unconditional associations between participant demographic characteristics, risk behaviors, and resistance to penicillin, tetracycline, and ceftriaxone. Variables shown in bold (p≤0.3) were retained for consideration in building the final multivariable model for each outcome.
(DOC)
