# Supplementary material for: Behavioral and Socioeconomic Risk Factors Associated with Probable Resistance to Ceftriaxone and Resistance to Penicillin and Tetracycline in Neisseria gonorrhoeae in Shanghai
Source: PLoS One. 2014 Feb 19;9(2):e89458. doi: 10.1371/journal.pone.0089458 (PMC3929748; doi:10.1371/journal.pone.0089458)
Supplement: Table S1 — P-values for the unconditional associations between participant demographic characteristics, risk behaviors, and resistance to penicillin, tetracycline, and ceftriaxone. Variables shown in bold (p≤0.3) were retained for consideration in building the final multivariable model for each outcome. (DOC) [file pone.0089458.s001.doc]

**Table S1. P-values for the unconditional associations between participant demographic characteristics, risk behaviors, and resistance to penicillin, tetracycline, and ceftriaxone***

|  | **Ceftriaxone**  **MIC>0.03** | **Ceftriaxone MIC>0.125** | **Penicillin Resistance** | **Penicillin Resistance Mechanism** | | | | **Tetracycline Resistance** | | **Tetracycline Resistance Mechanism** | | | | | | |  |
| --- | --- | --- | --- | --- | --- | --- | --- | --- | --- | --- | --- | --- | --- | --- | --- | --- | --- |
| **Variable** | *Decreased susceptibility* | *Decreased susceptibility* | *Resistant* | *Chromosomal* | | | *Plasmid* | *Resistant* | | *Chromosomal* | | | *Plasmid* | | | |  |
| Phase of the study (2 : 1) | 0.96 | 0.91 | **0.00** | **0.00** | | | **0.00** | 0.37 | | **0.01** | | | **0.00** | | | |  |
| Education (3 df) | **0.10** | 0.47 | **0.09** | **0.22** | | | | **0.01** | | **0.05** | | | | | | |  |
| Less than primary or   no education | Reference category for education | | | | | | | | | | | | | | |  | |
| Primary/middle school | 0.51 | 0.21 | 0.50 | 0.23 | 0.79 | | | 0.20 | | 0.29 | 0.31 | | | | | |  |
| High school | 0.84 | 0.12 | 0.82 | 0.64 | 0.56 | | | 0.65 | | 0.57 | 0.85 | | | | | |  |
| Above high school | 0.72 | 0.18 | 0.82 | 0.65 | 0.55 | | | 0.83 | | 0.68 | 0.97 | | | | | |  |
| Salary (4 df) | **0.23** | 0.94 | 0.61 | **0.06** | | | | **0.12** | **0.02** | | | | | | | | |
| <1300 Yuan | Reference category for salary | | | | | | | | | | | | | | |  | |
| 1300-2000 Yuan | 0.38 | 0.68 | 0.84 | 0.22 | 0.67 | | | 0.86 | 0.84 | | | 0.69 | | | | | |
| 2200-3500 Yuan | 0.06 | 0.98 | 0.46 | 0.52 | 0.45 | | | 0.06 | 0.51 | | | 0.01 | | | | | |
| 4000-5500 Yuan | 0.83 | 0.58 | 0.14 | 0.05 | 0.33 | | | 0.13 | 0.53 | | | 0.07 | | | | | |
| 6000-200000 Yuan | 0.09 | 0.58 | 0.51 | 0.65 | 0.41 | | | 0.95 | 0.28 | | | 0.55 | | | | | |
| Previous STI | 0.87 | 0.34 | 0.42 | 0.38 | 0.52 | | | **0.12** | 0.32 | | | 0.42 | | | | | |
| Previous bacterial STI | 0.88 | 0.75 | **0.22** | **0.26** | **0.27** | | | **0.14** | 0.52 | | | **0.09** | | | | | |
| Wash genitals before or after sex | 0.85 | 0.40 | **0.11** | **0.03** | 0.37 | | | 0.66 | 0.70 | | | 0.73 | | | | | |
| Wash genitals (refused) | 0.99 | 1.00 | 0.33 | 0.50 | **0.22** | | | 0.64 | 0.34 | | | 0.95 | | | | | |
| Take over the counter antibiotics | 0.47 | **0.28** | 0.67 | 0.56 | 0.77 | | | 0.64 | 0.72 | | | 0.67 | | | | | |
| Take over the counter antibiotics (refused) | 0.91 | **0.14** | **0.01** | **0.02** | **0.01** | | | 0.82 | 0.52 | | | 0.40 | | | | | |
| Age (4 df) | 0.76 | **0.04** | 0.95 | **0.18** | | | | 0.83 | 0.70 | | | | | | | | |
| 14-26 years | Reference category for age | | | | | | | | | | | | | | | | |
| 27-31 years | 0.56 | 0.77 | 0.88 | 0.88 | 0.78 | | | 0.41 | 0.36 | | | | | | 0.62 | | |
| 32-37 years | 0.44 | 0.77 | 0.91 | 0.32 | 0.48 | | | 0.99 | 0.59 | | | | | | 0.60 | | |
| 38-45 years | 0.71 | 0.29 | 0.42 | 0.52 | 0.43 | | | 0.90 | 0.41 | | | | | | 0.69 | | |
| 46-83 years | 0.47 | 0.02 | 0.78 | 0.42 | 0.92 | | | 0.46 | 0.41 | | | | | | 0.67 | | |
| Male: Female | **0.06** | **0.27** | 0.81 | 0.89 | 0.83 | | | **0.11** | **0.08** | | | | | 0.37 | | | |
| Use alcohol during sex | 0.58 | 0.85 | 0.48 | 0.62 | 0.42 | | | **0.01** | **0.03** | | | | | **0.05** | | | |
| Use drugs during sex | **0.22** | **0.24** | 0.46 | 0.57 | 0.53 | | | 0.51 | 0.68 | | | | | 0.50 | | | |
| Number of partners (3 df) | **0.20** | 0.43 | 0.47 | 0.33 | | | | 0.83 | 0.43 | | | | | | | | |
| One partner last 3 months | Reference category for number of partners | | | | | | | | | | | | | | | | |
| Two partners in last 3 months | 0.33 | 0.42 | 0.63 | 0.64 | | 0.72 | | 0.58 | 0.67 | | | | | 0.21 | | | |
| Three or more partners in last 3   months | 0.77 | 0.46 | 0.40 | 0.64 | | 0.28 | | 0.47 | 0.47 | | | | | 0.09 | | | |
| Refused number of partners in   last 3 months | 0.10 | 0.40 | 0.33 | 0.80 | | 0.11 | | 0.81 | 0.80 | | | | | 0.88 | | | |

*Reference category for all outcomes was susceptibility as determined by MICs.

Variables shown in bold (p≤0.3) were retained for consideration in building the final multivariable model for each outcome.
